# The General Practitioner and the Railroads†Read before Jefferson County Medical Society, August meeting.

**Published:** 1906-09

**Authors:** P. W. Beckman

**Affiliations:** Beaumont, Texas


For Texas Medical Journal.

### The General Practitioner and the Railroads.†

BY P. W. BECKMAN, M. D., BEAUMONT, TEXAS.

Along the line of recent agitation for the betterment of the financial status of the medical profession, I should like to make a few pertinent observations from the standpoint of the general practitioner with regard to railroad practice, viz.: the attitude of the railroad company to the large mass of the medical profession.

First, the system by which the railroad company manages to obtain the services of local surgeons along the road for practically nothing, or for totally inadequate compensation, advantage being taken of some young man who wishes to gain prestige thereby, or who thinks that it will give him an entree to surgery or some hospital, and also to make the acquaintance of the families of railroad employes; or else some older man is holding on to the unremunerative position for fear that he is missing, or might miss, something, or to keep some younger man from getting practice.

This is certainly not charity, for these men are able-bodied men and make good salaries, and are more able to pay a doctor than two-thirds of the people who pay us, and if the per capita assessment is not sufficient to pay the doctor a half-way decent fee for his work, it should be made so, and no part of it should be diverted to the settlement of damage claims for which railroads are legally

---

*Prof. Elias J. Beall, M. D., Emeritus Professor Practice of Medicine, University of Fort Worth. Editor.

†Read before Jefferson County Medical Society, August meeting.

liable, but it should go towards the payment of physicians for services for which they are entitled to be paid.

It is certainly a case of the medical profession "getting worked" by the railroad companies, by taking advantage of conditions and professional jealousies and offering indirect emoluments to obtain work for practically nothing. While harping about reduction in insurance fees, it might be well to give the foregoing some thought. The medical profession should raise its standard of honesty to such a point as not to recognize such indirect emoluments as compensation, but should work for the good of the whole instead of allowing themselves to be taken advantage of in that way. The system is certainly inimical to the best interests of the profession. These remarks do not apply to chief surgeons, as I believe they are pretty well paid.

Second, the treatment of the general profession by some of the various chief surgeons, as an example I will cite: When I first located in this place, I was called on the night of a severe rainstorm about three years ago; was told that neither of the local railroad surgeons was accessible and that a man was seriously injured. I was obliged to wade through water almost to my waist, in the dark and rain, as this was the night of our severe rain-storm here about three years ago. I found the patient had fallen off a moving freight train; was unable to walk or move about in bed without causing excruciating pains; hip very much swollen and bruised, also bruised about the back; retention of urine the following morning; least movement of limbs caused very severe pains and was desisted from on account of possibility of impacted fracture of the neck of the femur. A careful examination was made and an opinion expressed as to the nature of the injuries. Hypodermic, one-half morphia, was required to quiet patient; was advised to get railroad surgeon in the morning. At his request I called again the following morning, more particularly to satisfy myself as to the exact nature of injuries, and the next morning I again called at the solicitation of the local surgeon, and we examined the man together, and I resigned the case to him.

A bill for $9 was sent to the chief surgeon, of which no cognizance was taken; a second bill was sent through local surgeon with explanation of circumstances under which services were rendered, and answer was received that one visit would be paid for. I then wrote this chief surgeon that I considered that $25 would really have been a very small fee for making an examination at night, of that character of an injury, and stating whether or not fracture of femur or injury of vertebra existed, and instituting treatment accordingly, also the conditions under which services were rendered. I stated that the transaction was small, unprofessional and shortsighted, and not conducive to gain the good will of the profession, or words to that effect.

This is only one instance of dealings of chief surgeons with local physicians.

Further, I have any number of times been approached by claim agents, and have given them written statements, from case records, of cases that had left my jurisdiction, and at no time have I ever been paid for my time. It would certainly only be just that pay should be tendered for such work. It really involves an opinion which ought to be worth as much as a consultation. At times, claim agents ask leading questions in an insinuating manner, which is altogether out of place. Railroad companies frequently or generally evade payment for physicians' services, on the grounds that party calling for physician was not authorized to do so. It is true that some of these bills may be unjust and that they can not be expected to pay a physician in ordinary cases, where they have a man employed and he is accessible.

As far as accepting contingent fees, I believe that is an extreme exception. I do not believe that any physician waives the right of his fee in case no damage is awarded, and that it is a very rare exception that he puts his fee on anything like a percentage basis. The fee is only contingent in so far that these people are frequently unable to pay a doctor, and in those cases no fee is collected unless damage is awarded, and I believe that in nine out of ten physicians, this does not influence them towards giving untruthful testimony.

So far as traumatic neurasthenias and hysterias are concerned, they will continue to be an existent evil, for they are in some cases a greater cause of invalidism than actual injuries, and it is very hard to definitely settle extent of disability and permanence, and they usually get worse instead of better with protracted litigation. Concerning the remarks that these neuroses are not met with following nervous shock in battle, will say that the character of injuries in battle is totally different from wrenches or contusions to back and head; the incentive in the army to obtain a discharge on disability certificates and subsequent pensions, if anything, is greater than a railway damage award.

On account of attempted brevity, some of the remarks in this paper may be misconstrued. It is not intended to convey the idea that the existing conditions are a justification for dishonesty or aggravating litigation against railroad companies, but that there are two sides to the story, and that it would be only just if railroad companies and chief surgeons would show a little more courtesy, in some of these matters, to the general profession. The latter, in some cases, being men of only mediocre ability, and the show of arrogance and attempts to impute dishonesty are a little out of place, when they themselves show an unloyal spirit and are fostering a system which is injurious to the profession. We do not blame them for holding good jobs, but think they should come out of the "amen" corner a little bit, and put off some of their air of saintliness.

